# Nucleolar Organization, Ribosomal DNA Array Stability, and Acrocentric Chromosome Integrity Are Linked to Telomere Function

**DOI:** 10.1371/journal.pone.0092432

**Published:** 2014-03-24

**Authors:** Kaitlin M. Stimpson, Lori L. Sullivan, Molly E. Kuo, Beth A. Sullivan

**Affiliations:** 1 Department of Molecular Genetics and Microbiology, Duke University Medical Center, Durham, North Carolina, United States of America; 2 Institute for Genome Sciences & Policy, Duke University, Durham, North Carolina, United States of America; 3 Division of Human Genetics, Duke University, Durham, North Carolina, United States of America; Texas A&M University, United States of America

## Abstract

The short arms of the ten acrocentric human chromosomes share several repetitive DNAs, including ribosomal RNA genes (rDNA). The rDNA arrays correspond to nucleolar organizing regions that coalesce each cell cycle to form the nucleolus. Telomere disruption by expressing a mutant version of telomere binding protein TRF2 (dnTRF2) causes non-random acrocentric fusions, as well as large-scale nucleolar defects. The mechanisms responsible for acrocentric chromosome sensitivity to dysfunctional telomeres are unclear. In this study, we show that TRF2 normally associates with the nucleolus and rDNA. However, when telomeres are crippled by dnTRF2 or RNAi knockdown of TRF2, gross nucleolar and chromosomal changes occur. We used the controllable dnTRF2 system to precisely dissect the timing and progression of nucleolar and chromosomal instability induced by telomere dysfunction, demonstrating that nucleolar changes precede the DNA damage and morphological changes that occur at acrocentric short arms. The rDNA repeat arrays on the short arms decondense, and are coated by RNA polymerase I transcription binding factor UBF, physically linking acrocentrics to one another as they become fusogenic. These results highlight the importance of telomere function in nucleolar stability and structural integrity of acrocentric chromosomes, particularly the rDNA arrays. Telomeric stress is widely accepted to cause DNA damage at chromosome ends, but our findings suggest that it also disrupts chromosome structure beyond the telomere region, specifically within the rDNA arrays located on acrocentric chromosomes. These results have relevance for Robertsonian translocation formation in humans and mechanisms by which acrocentric-acrocentric fusions are promoted by DNA damage and repair.

## Introduction

Human telomeres are tandem double-stranded TTAGGG repeats located at the termini of chromosomes where the assembled protein complex acts as a protective cap, preventing degradation of the telomere repeats, end-to-end fusions, and recognition of single-stranded and double-stranded ends as damaged DNA [1]. Dysfunctional telomeres activate DNA damage checkpoints, trigger repair/recombination machinery, and produce genomic rearrangements as a result of fusion events [2]. TRF2 (TTAGGG repeat binding factor 2) is a key member of the telomere protein complex, binding double-stranded DNA at telomeres, and facilitating topological changes in the telomeric DNA and T-loop assembly [3]–[5]. TRF2 provides a protective capping function at the telomere, suppressing DNA damage recognition and non-homologous end joining [3], [4], [6]–[8].

Disrupting telomere function by expressing the mutant telomere protein TRF2^ΔBΔM^ (hereafter called dnTRF2) induces formation of *de novo* dicentric human chromosomes [9], [10]. We previously showed that chromosome fusions occur non-randomly after short-term dnTRF2 expression (36 hours), with nearly 80% occurring between the short arms of the *Homo sapiens* acrocentric chromosomes 13, 14, 15, 21, and 22 (HSA13, HSA14, HSA15, HSA21, HSA22) [9]. When they form naturally in humans, acrocentric fusions are called Robertsonian translocations and represent the most common human chromosomal rearrangement (1 in 1000 live births) [11]. The reversible telomere disruption assay models a prevalent structural human chromosome abnormality, and provides a system to probe the molecular basis for formation and stability of acrocentric fusions.

The mechanism driving Robertsonian translocation formation is thought to depend on genomic organization of acrocentric chromosomes. All 10 acrocentric short arms share several highly similar or identical blocks of repetitive DNA, including satellite III (sat III) and beta satellite [12], [13]. In addition, approximately 400 copies of the 43 kb ribosomal DNA (rDNA) cassette are distributed among the acrocentric short arms, existing as clusters called nucleolus organizing regions (NORs) [14]. The nucleolus is assembled around the ribosomal RNA genes (NORs) each cell cycle. Its main function is to produce ribosome subunits. After exit from mitosis, numerous mini-nucleoli are formed around actively transcribing NORs [14]–[16]. The mini-nucleoli fuse to form larger nucleoli [17]–[19], thereby bringing the NORs of multiple acrocentrics into close proximity [20]–[22]. The transcription factor UBF binds to rDNA arrays and sequesters NORs - and thus, the acrocentric chromosomes - to common nuclear subdomains [23]–[25]. Acrocentric fusions are proposed to occur via incomplete homologous or non-homologous recombination between short arm repeats or through repair of short arm DNA damage that is corrected using a similar short arm DNA sequence on a nearby non-homologous acrocentric.

The nucleolus harbors a diverse set of proteins [26], suggesting that it functions in roles beyond ribosome biogenesis. With a dynamic and diverse protein pool, nucleoli may also be a focal point for organizing or amplifying the stress response. Nucleolar architecture and rDNA transcription change in response to cellular stresses such as DNA damage, viral infection, and temperature variation [27]. Here we describe a dramatic change in nucleolar morphology and acrocentric short arm DNA organization that occurs when telomere function is disrupted. Our results suggest that one response is a change in nucleolar structure and under-condensation of NORs punctuated by UBF protein bridges that physically tether acrocentric short arms and promote acrocentric fusion.

## Materials and Methods

### Cell culture

The human HT1080 cell line derivative called HTC75T19 (T19) expresses a truncated allele of TRF2 (dnTRF2) under the control of a tetracycline/doxycycline-sensitive promoter [10]. HT1080 cells, 293T cells, and the T19 clonal cell line were cultured in minimum essential medium alpha (Invitrogen) supplemented with 10% FBS (HyClone and Cellgro) and antibiotics (Invitrogen). The T19 cell line media also contained 5 mM glucose and 100 ng/mL doxycycline hyclate (Fluka). T19 dox-inducible cells were induced by washing 3 times in phosphate buffered saline (PBS) before incubation in tetracycline/doxycycline free media. Cells were incubated with 33 μg/mL zeocin (Invitrogen) for 36 hours before metaphase chromosomes were isolated. For Actinomycin D treatments, T19 cells were treated with 50 ng/mL (Sigma) for 30 minutes, 3 hours, or 4 hours before RNA isolation or 5FU treatment. T19 cells growing on chamber slides were treated with 1 mM 5FU (Sigma) for 30 minutes.

### Nucleolar isolation

Nucleoli were isolated from HT1080, uninduced T19, and 30- or 32-hour induced T19 cells using a previously described method [28] subsequently modified by the Lamond lab [29]. Briefly, ∼10^8^ cells were harvested, washed with cold PBS, resuspended in 5–7 mL of buffer (10 mM HEPES, 1.5 mM MgCl_2_, 10 mM KCl, 0.5 mM dithiothreitol), and dounce homogenized on ice (tight pestle) until nuclei were released. Nuclei were gently pelleted, resuspended in 3 mL of Sucrose 1 (0.25 M sucrose, 10 mM MgCl_2_), and layered over Sucrose 2 (0.35 M sucrose, 0.5 mM MgCl_2_) before centrifugation at 1430×*g* for 5 minutes. The pellet was resuspended in 3 mL Sucrose 2 before sonication with a Misonix Microson XL 2000 sonicator at power 10 with 5 second bursts on ice until nuclei were broken open and nucleoli were distinct. The sonicated solution was layered over 3 mL Sucrose 3 (0.88 M sucrose, 0.5 mM MgCl_2_) and centrifuged at 2800×*g* for 10 minutes before resuspension in Sucrose 2.

### Immunofluorescence – nuclei

HT1080 and T19 cells were seeded on Fisher SuperFrost/Plus slides. 5FU treated cells were briefly extracted with PBS+0.2% Tween 20 before fixation. Cells were fixed with 4% PFA in PBS for 10 minutes and permeabilized in PBS+0.1–0.25% Tween 20 for 30 minutes to 1 hour. Slides were incubated at 4°C overnight in blocking buffer with antibodies at dilutions listed below.

### Immunofluorescence - metaphase chromosomes

Spreads were prepared as previously described [30]. Briefly, confluent cells were incubated with 0.05 μg/mL colcemid (Gibco/Invitrogen) for 1 hour and swelled in hypotonic buffer (1∶1∶1 v/v/v 75 mM KCl: 0.8% Na citrate: H_2_O) for 10 minutes. The cell concentration for extended or elongated chromosomes was 3.5×10^4^ cells/mL. Swelled cells were subject to centrifugation onto slides using a Shandon Cytospin 4. Chromosomes were fixed in 4% PFA in PBS and extracted in PBS+0.1% Tween 20 before incubation with antibody solution overnight at 4°C. For RNase A/H treatments, slides were treated with 100 μg/mL RNase A and RNase H (NEB) at 37°C for 20 minutes prior to incubation with antibody solution.

### Immunofluorescence - isolated nucleoli

Nucleoli suspended in Sucrose 2 were spread across slides and fixed with 4% PFA in PBS for 10 minutes, rinsed in PBS, and incubated with antibodies overnight at 4°C.

### Antibodies used

Antibodies included fibrillarin 1∶1000 (Abcam ab18380 or ab5821), CENP-A 1∶500 (Abcam 13939 or Upstate 07-574), TRF2 1∶200 (Imgenex IMG-124A or Novus NB110-57130), Ki67 antigen 1∶500 (Novocastra Laboratories Ltd.), H2AX-p 1∶300 (Millipore 05-636 or Abcam ab2893), ATM-p Ser1981 1∶250 (Cell Signaling 5883), Chk2-p Thr68 1∶250 (Cell Signaling 2661), SMC2 1∶300 (Cell Signaling 5394), SMC4 1∶300 (Cell Signaling 5547), goat polyclonal to DDDDK (FLAG) tag 1∶500 (Abcam ab1257), UBF 1∶150 (Santa Cruz H-300), and anti-BrdU 1∶400 (Sigma clone BU-33). Primary antibodies were detected using anti-mouse, anti-rabbit, or anti-goat secondary antibodies conjugated to Alexa Fluor 488, 594, 647 (Molecular Probes), FITC, Cy3, or Cy5 (Jackson Immunoresearch, Inc.).

### Fixed chromosome and nuclei isolation

Confluent cells were harvested according to standard methods with hypotonic buffer (1∶1∶1 v/v/v 75 mM KCl: 0.8% Na citrate: H_2_O), fixed (3∶1 v/v methanol: acetic acid), and dropped onto clean glass microscope slides.

### Probe preparation

pTRS-47 (sat III) and pTRS-63 (sat III) plasmids were generously provided by Professor Andy Choo (Melbourne, Australia). The beta satellite repeats were detected using the pβ4 plasmid [31]. The plasmids for 18 s rDNA, 13/21 alpha satellite, and 14/22 alpha satellite were constructed from previously described PCR primers using the TOPO TA Cloning Kit (Invitrogen) and One Shot TOP10 Competent Cells according to manufacturer's specifications [32]–[34]. A cosmid containing the 43 kb rDNA repeat was used as a probe for the entire rDNA region (a kind gift of Brian McStay, NUI, Galway). Plasmids and cosmids were labeled with biotin-16-dUTP, digoxygenin-11-dUTP (Roche), Molecular Probes ChromaTide Alexa Fluor 488-5-dUTP or Alexa Fluor 568-5-dUTP (Invitrogen) by nick-translation according to Molecular Probes. Whole chromosome paints were made using isolated DNA from the Human/Rodent Somatic Cell Hybrids Mapping Panel #2 (Coriell). Chromosome specific DNA was amplified using degenerate-oligonucleotide-primed PCR (DOP-PCR) and labeled with biotin-16-dUTP or digoxygenin-11-dUTP. Telomere repeats were detected with FITC-conjugated Peptide Nucleic Acid (PNA) probe (C_3_TA_2_)_3_ (Biosynthesis). CENP-B box sequences were detected with a biotin-labeled Peptide Nucleic Acid (PNA) probe.

### Fluorescence in situ hybridization (FISH)

FISH and immunostaining-FISH were performed as previously described [30]. Probes used recognized beta satellite (pβ4) [12], satellite III (pTRS-47 and pTRS-63) [35], and rDNA/NORs (PAC probe RP5-1174A5) (CHORI BAC/PAC Resource). All probes were labeled with biotin-16-dUTP or AlexaFluor-dUTP by nick translation. Methanol:acetic acid fixed nuclei and chromosomes were denatured in 70% formamide/2X SSC pH 7 at 70°C for 1 minute. Probe was denatured at the same temperature for 8–10 minutes in hybridization mixture. PFA fixed nuclei, metaphase chromosomes, and nucleoli were co-denatured with probe on a hot plate at 80°C for 2–3 minutes. The hybridization solution consisted of 50–70% formamide, 2X SSC, 1% Tween 20, and 10% dextran sulfate. Probe was hybridized to DNA under a sealed coverslip overnight in a humidified chamber at 37°C. Slides were washed in 50–70% formamide/2X SSC at 25–42°C followed by washes in 2X SSC at 25–37°C. Probe was detected by 2 hour incubation at room temperature with Cy3-, Cy5-, or Alexa Fluor 488-conjugated anti-digoxin or avidin secondary antibodies (Jackson ImmunoResearch and Molecular Probes) and washed in PBS+0.1% Tween 20 before mounting in Vectashield (Vector Labs, Burlingame, CA) containing 2 μg/mL DAPI.

### RNA FISH

T19 cells were seeded on Fisher SuperFrost/Plus slides, washed with PBS, incubated with 100 μg/ml RNase A for 10 minutes, incubated with PBS+0.2% TritonX for 1 minute, fixed in 4% PFA in PBS for 10 minutes, and permeabilized in PBS+0.5% TritonX for 5 minutes. Slides were incubated in 20% glycerol for 20 minutes before submersion in liquid nitrogen for 30 seconds. Control slides were then treated with 100 μg/mL RNase A for 1 hour at 37° and RNase H (NEB) for 10 minutes. All slides were lightly denatured at 70°C in 40% formamide/2X SSC for 45 seconds, dehydrated in an ethanol series, and hybridized with probe in 50% formamide hybridization buffer (2–3 ng probe/mL buffer) for at least 1 hour. Washes were performed at 37°C in 50% formamide/2X SSC for 15 minutes and in 2X SSC for 10 minutes. 18S probes were direct labeled (IDT) as described [36].

### Knockdown of TRF2 by short hairpin RNA expression

293T cells were used to make retrovirus derived from pSUPER-Retro-puro empty vector and pSUPER-Retro-puro vector containing small hairpin RNA for TRF2 (kind gift of Chris Counter, Duke University). 293T cells were transfected with the retroviral and packaging plasmid using FuGene 6 (Roche). Filtered media containing the retrovirus along with 5 μg/mL Polybrene (Millipore) were added to HT1080 cells for infection. Infected HT1080 cells were selected with 1 μg/mL Puromycin (MP Biomedical) and polyclonal populations were collected at 11 and 15 days for immunoblot and immunofluorescence.

### Immunoblot

Cell pellet samples were lysed and boiled for 5–10 minutes with XT or Laemelli Sample Buffer (Bio Rad) with reducing agent. UV-treated cells were collected 2 hours after UV irradiation (20 J/m^2^) and immediately lysed and boiled. Kaleidoscope protein markers (Bio Rad) were used as standards. Samples were loaded onto a 4–12% Tris-HCl polyacrylamide gel (Bio Rad) or 4–12% Criterion XT Bis-Tris gel (Bio Rad) and transferred to a polyvinylidene fluoride membrane (Millipore). The membranes were incubated with rabbit polyclonal TRF2 antibody 1∶1000 (Novus NB110-57130), Chk2-p Thr68 1∶500 (Cell Signaling 2661), mouse monoclonal to fibrillarin 1∶750 (Abcam ab18380), H2AX-p 1∶1000 (Cell Signaling 9718), or beta actin 1∶2000 (Abcam ab6276) in blocking buffer. The membrane was incubated with the species-specific horseradish peroxidase-conjugated secondary antibody 1∶5000 (Abcam anti-mouse ab6829 or anti-rabbit ab6902) and visualized using an ECL system.

### Chromatin immunoprecipitation

ChIP assays with the MAGnify Chromatin Immunoprecipitation System (Invitrogen) were performed according to manufacturer's protocol. Confluent cells were crosslinked in 1% formaldehyde for 10 minutes, sheared to 100–700 base pair fragments with a Misonix Microson XL 2000 at power level 10 repeating a 5 seconds on/5 seconds off cycle 30–35 times on ice. Invitrogen Dynabeads were incubated with the following antibodies for 1–2 hours at 4°C: 1 μL IgG rabbit (Invitrogen), 2 μL UBF (Santa Cruz 9131), 4–5 μL TRF2 (Novus NB110-57130), 2 μL H3K4me2 (Abcam ab7766), and 1.5 μL H3K9me3 (Abcam ab8898). 150,000 cells were used in each immunoprecipitation (IP) and incubated with the antibody/Dynabeads for 2–3 hours at 4°C. Beads were washed, chromatin reverse crosslinked, and DNA eluted according to the MAGnify ChIP protocol. ChIPs were repeated at least 3 times for each modification.

### PCR

Eluted IP DNA was amplified for semi-quantitative PCR using a Bio-Rad myCycler or S1000 Thermal Cycler. Primer sequences included: rDNA primers u18S, 18S, and intergenic spacer (IGS) corresponding to H1, H4, and H18 respectively [37], beta satellite distal [38], and degenerate alpha satellite [39]. Two microliters of IP DNA was used in each 15 μL PCR reaction in duplicate. Amplification products were run on a 1.5% agarose gel. Band intensity was quantified using ImageJ software (http://rsb.info.nih.gov/). Relative enrichments of histone modifications or chromatin binding proteins were calculated as described using the following formula: [(IP−Mock)/(Input−Mock)]_Query_/[(IP−Mock)/(Input−Mock)]_Control site/Normalizer_ [40].

### qRT-PCR

cDNA was collected from uninduced T19 control, 12-, 24-, 48-, and 72-hour dnTRF2-expressing cells, serum starved cells, and cells treated with Actinomycin D for 30 minutes and 3 hours. cDNA was isolated using Qiagen's FastLane Cell cDNA kit (Cat. 215011). One confluent T25 flask per sample was used and the manufacturer's protocol was followed. qRT-PCRs were performed using Qiagen's QuantiFast SYBR Green PCR kit (Cat 204052) for GAPDH (primers: 5′-CTCATGACCACAGTCCATGCC-3′, 5′-GCCATCCACAGTCTTCTGGGT-3′) [41], 45S pre-ribosomal RNA (primers: 5′-CTCCGTTATGGTAGCGCTGC-3′, 5′-GCGGAACCCTCGCTTCTC-3′), and 28S ribosomal RNA (primers: 5′-CGACGACCCATTCGAACGTCT-3′, 5′-CTCTCCGGAATCGAACCCTGA-3′) [42]. Each primer pair was optimized (e.g. performing standard dilute and melt curve analyses) using HT1080 cDNA prior to performing qRT-PCR on the samples. Each sample was performed in triplicate within the respective run. Ct values (triplicates) were averaged and the SEM was calculated. Fold change was calculated using the following formulas:

ΔCt =  target Ct - endogenous Ct (i.e. GAPDH)

ΔΔCt =  ΔCt sample - ΔCt Calibrator (i.e. T19 control)

Fold Change (Relative Expression)  = 2^ΔΔCt^

### Microscopy

Images were acquired on an inverted Olympus IX-71 attached to the Deltavision RT or Core imaging system (Applied Imaging, Inc) and Photometrics CoolSNAP HQ CCD camera. All objectives used were oil objectives from Olympus and included: 40× (UAPO 1.35 NA) and 60× (PLAN-APO, 1.40 NA) for metaphase chromosomes, and 60× or 100× (PLAN APO 1.40 NA) for isolated nucleoli and nuclei. Images were acquired using Deltavision SoftWoRx Resolve 3D capture program and collected as a stack of 0.1–0.5 micrometer increments in the z-axis. Images consisted of 1–20 sections (0.5–5 micrometers), depending on the fixation technique. Images were deconvolved using the conservative algorithm with 10 iterations, and stacked images were viewed by quick projection. Projections were converted to Adobe Photoshop for viewing and analysis.

### Image analyses

Digital images were captured using an epifluorescence microscope with no signal reaching pixel saturation. Using IPLab/iVision, pseudo-colored three-color (RGB) images were separated into individual wavelengths for the blue (457 nm), green (488 nm), and red (568 nm) channels. Pearson coefficient values were obtained by using the SoftWoRx co-localization tool. Degree of co-localization of the signals from two wavelengths (TRF2 and SMC4) was visualized on a scatterplot of pixel intensities. The relative amount of SMC4 on metaphase chromosomes was quantitiated using a custom IPLab/iVision (BioVision Technologies) script that measures arbitrary fluorescence intensities along the length of a chromosome. The region of interest (ROI) was defined by manually tracing a chromatid and arbitrary fluorescence values along the ROI were plotted for each wavelength as a contiguous line plot. For each chromosome immunostained for SMC4 and hybridized with an rDNA FISH probe, a line was manually drawn along the chromatid from the end of the p arm (0 on x-axis of resulting graph), through the NOR (rDNA) and centromere, to the telomere of the q arm (∼150+ on x-axis of the resulting graph). Graphical presentation of the percentage of chromosomes exhibiting specific patterns of SMC4 localization at rDNA was achieved using Kaleidagraph. Categories were defined as: “co-localization” (rDNA and condensin peaks coincided or nearly overlapped), “partial co-localization” (∼50% of the condensin peak overlapped with the rDNA peak), or “no co-localization” (rDNA peak fell between condensin peaks or in a region comparable to the low signals across the chromosome). The frequency of UBF/rDNA connections was determined by collecting all measurable metaphases on at least two slides from two independent cell collections and cytospin experiments.

### Statistics

For comparisons between the proportions of abnormal and normal nuclei and FISH signals in Figures S1–S3, contingency tables were used and, if applicable, correction for multiple testing using the Marascuilo procedure was applied (http://www.stattools.net/Multiprop_Pgm.php) and verified with Bonferroni's correction. Student's t-test was used for analyses of control and dnTRF2 ChIP enrichments, control and dnTRF2 nucleoli immunofluorescence intensity, control and dnTRF2 Pearson coefficients, and qRT-PCR data.

## Results

We previously showed that transient telomere disruption caused by expression of inducible, dominant-negative mutant allele of the telomere protein TRF2 (dnTRF2) for 36–48 hours perturbs the nucleolus and produces acrocentric fusions [9], [10]. The prior study focused on dicentric formation and fate and centromere function. The molecular basis for nucleolar disruption or acrocentric sensitivity to telomere dysfunction induced by dnTRF2 was not explored. Here, we sought to address this issue, specifically focusing on the temporal events leading to nucleolar disruption and non-random acrocentric chromosome fusion. To verify that our observations were not restricted to the dnTRF2 system, we used additional approaches to cripple telomeres and verify effects on nucleolar morphology and acrocentric stability.

**Figure 1:**
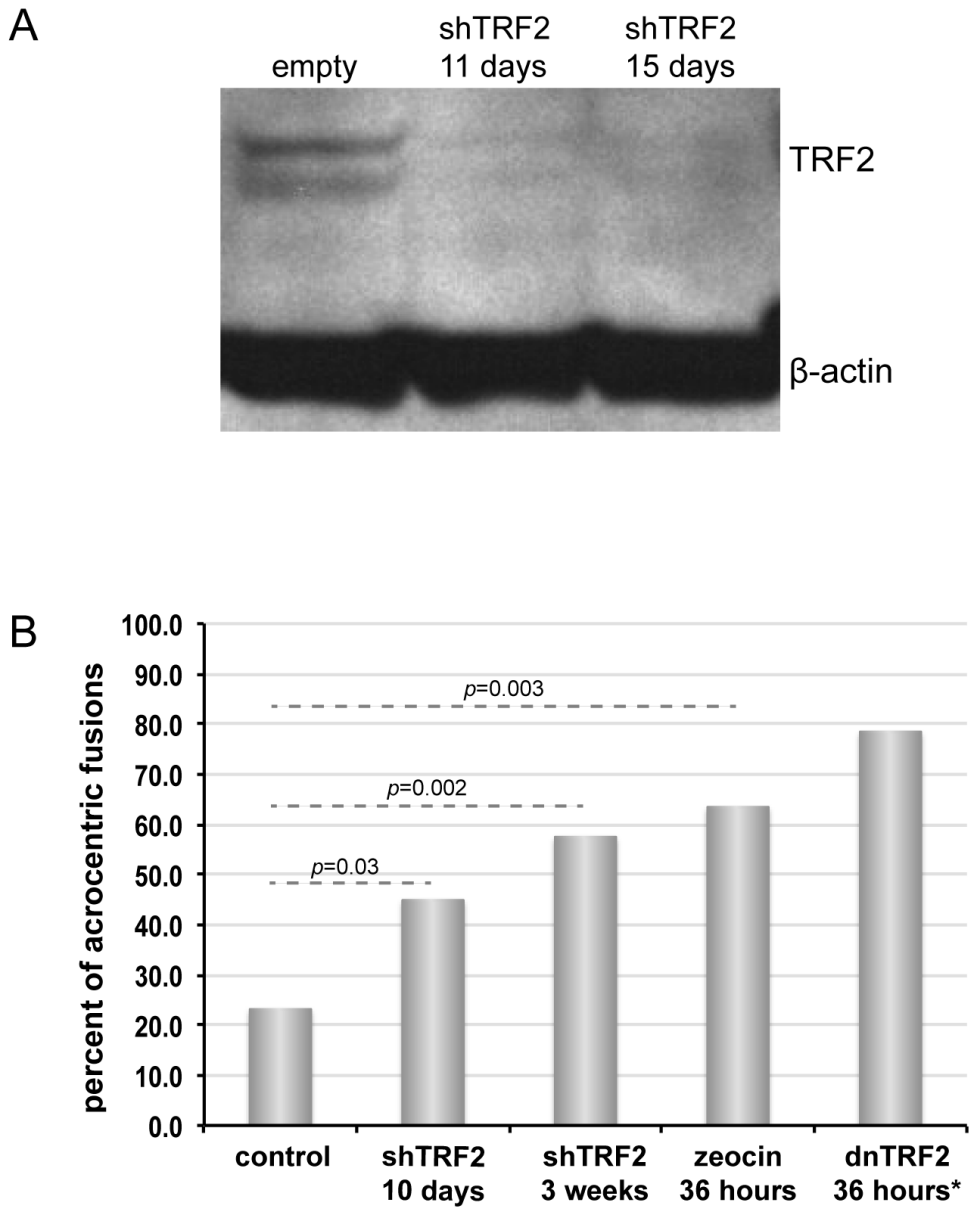
Non-random acrocentric fusion when telomeres are disrupted by various approaches. (A) Immunoblot of HT1080 whole cell lysates selected for empty vector or shTRF2 retroviral vector for 11 or 15 days with puromycin. Blot shows TRF2 protein as a doublet and β-actin as a loading control. (B) Acrocentric fusions are non-randomly induced in HT1080 cells expressing a retroviral shTRF2 construct for 10 days and 3 weeks. In addition, treatment of cells with the double-strand break inducer zeocin also results in high numbers of acrocentric fusions. asterisk (*) in graph legend denotes observations from dnTRF2 expression previously reported in [9].

First, TRF2 was depleted by expression of a retroviral short hairpin RNAi construct. At both 10 days and 2 weeks of shTRF2 expression, Western blotting and immunostaining showed that TRF2 had been largely depleted (Figure 1A, Figure S1A, B). The nucleolus also showed a disordered appearance matching what was previously observed in dnTRF2 cells (Figure S1A, S1C) [9]. An elevated number of acrocentric fusions were observed compared to control cells (Figure 1B). The number of acrocentric fusions increased the longer that shTRF2 was expressed.

In the second approach, we treated cells with zeocin, a DNA-damaging agent that induces double-strand breaks throughout the genome, including at telomeres [43]. After zeocin treatment for 36 hours, nearly 65% of cells contained acrocentric fusions (Figure 1B). This frequency was similar to that observed for acrocentric fusions induced by dnTRF2 (Figure 1B) [9]. These experiments supported our previous findings that human acrocentric chromosomes are hypersensitive to telomere disruption and DNA damage. They also suggested that non-random acrocentric fusion is one consequence of telomere damage and is not restricted to the dnTRF2 assay.

### Altered nucleolar structure is coincident with dnTRF2 expression

**Figure 2:**
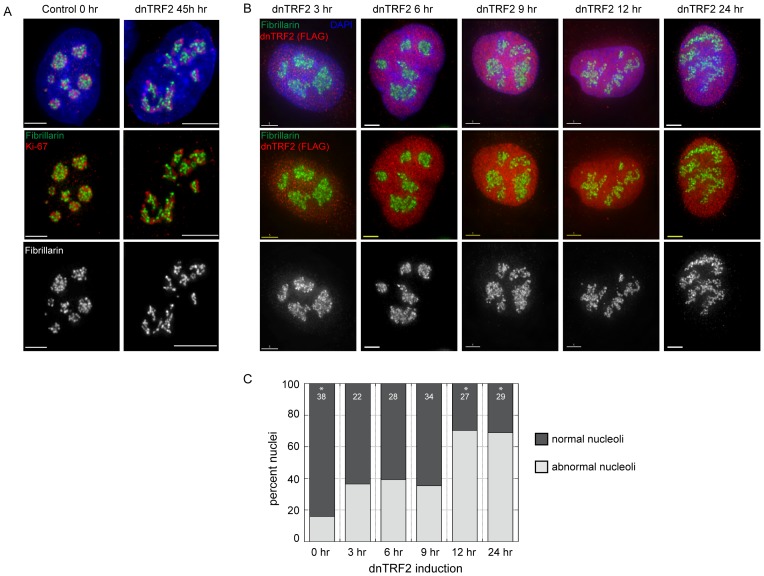
Timing of nucleolar protein disruption with increasing dnTRF2 expression. (A) Immunostaining of 3D-preserved whole nuclei with nucleolar protein fibrillarin (green) and Ki-67 (red) in control and 45 hour dnTRF2 nuclei show that nucleolar morphologies changes with increased dnTRF2 expression and telomere dysfunction. (B) Induced T19 (dnTRF2-expressing) cells were analyzed at intervals over a 24-hour period using immunofluorescence with antibodies specific to fibrillarin and FLAG (to detect FLAG-tagged dnTRF2 protein - red). Nucleolar changed from a normal spherical shape to less condensed structures resembling nucleolar necklaces. (C) Quantitation of the percent of nuclei showing visibly abnormal nucleolar staining over the timecourse. Abnormal morphology (decondensed, unraveled) of nucleoli (light grey) increased as dnTRF2 was expressed for longer periods. The number of nuclei examined at each timepoint is indicated at the top of each stacked bar. After dnTRF2 expression for 24 hours, there was a statistically significant increase (asterisk) in the proportion of abnormal nucleoli compared to control cells. Scale bars equal 5 micrometers, hr =  hour.

All three strategies (TRF2 knockdown, zeocin treatment, and dnTRF expression) produced nucleolar and chromosome defects, but the dnTRF2 system provided the most controllable method for generating the highest number of acrocentric fusions and altered nucleoli. It was used in subsequent experiments. We established that both nucleolar organization, denoted by fibrillarin, Ki-67, and B23 immunostaining, appears abnormal after 36–40 hours of dnTRF2 expression [9] (Figure 2A, Figure S2). We wished to determine which occurred first: nucleolar disassembly or acrocentric short arm instability. To establish detailed timing of these events, T19 (dnTRF2) cells were induced and monitored at intervals over a 36 hour period followed by fibrillarin immunostaining (Figure 2B). By 9 hours, 35% (12/34) of nuclei exhibited abnormal fibrillarin staining. Abnormal staining was determined visually in that compact nucleoli appeared unraveled, occupying an increased area of the nucleus (compare control nucleoli in Figure 2A with Figure 2B). From 12 hours on, the abnormal morphology increased, peaking in 70% of cells (19/27) by 24 hours (Figure 2C). The abnormal nucleolar phenotype was not due simply to effects of the dominant-negative protein, since nucleoli were similarly disrupted when TRF2 was knocked down using a short hairpin RNAi construct (Figure S1A, S1C).

### Acrocentric short arm DNA reorganization during dnTRF2 expression

**Figure 3:**
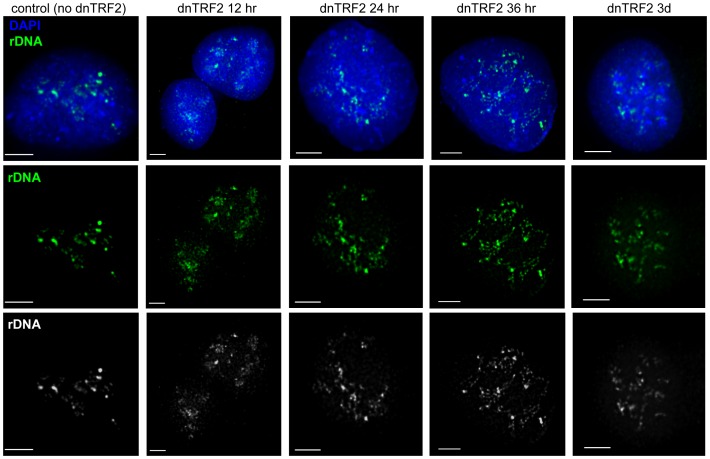
dnTRF2 expression correlates with rDNA repeat array dispersion. The rDNA arrays are located on the short arms of the 5 pairs of acrocentric chromosomes. FISH on RNase-treated nuclei hybridized with an rDNA (green) PAC probe showed that rDNA, normally appearing as multiple punctate foci in the nucleus, becomes more diffuse with increased dnTRF2 expression. The T19 (dnTRF2) cell line contains ∼18 acrocentric chromosomes. Multiple short arms normally converge in the nucleus, so each foci can contain more than rDNA regions from more than one acrocentric chromosome. With increased dnTRF2 expression and telomere dysfunction, the bright foci were reduced, instead appearing as dotted or beaded tracks of fluorescent signals stretching throughout the nucleus. Pseudo-colored and gray-scale single channel images for rDNA are shown below the merged images. Scale bars equal 5 micrometers.

Progression of acrocentric short arm DNA disruption was visualized using fluorescence *in situ* hybridization (FISH) with DNA probes that recognized each of the distinct repetitive arrays (rDNA, beta satellite, satellite III, and alpha satellite DNA) on the acrocentric short arms (Figure 3, Figure S3). Starting at 24 hours after dnTRF2 expression, rDNA FISH signals appeared more scattered throughout the nucleus, contrasting with the punctate clustered, and bright foci observed in control cells (Figure 3, Figure S3D). By 36 hours, the punctate FISH signals were smaller and dotted “tracks” of FISH signal were apparent. By three days, the concentrated rDNA FISH signals were less apparent and more diffuse in appearance. Satellite III FISH signals also became dispersed in the presence of dnTRF2 (Figure S3B and S3D). Centromeres, detected using an alpha satellite DNA probe, and beta satellite DNA located on the proximal short arm remained relatively punctate in both control and dnTRF2 nuclei (Figure S3A, S3C). Acrocentric short arm DNA instability occurred shortly after nucleolar changes (36 hours versus 24 hours). These results suggest that acrocentric short arm structure, and particularly the block of rDNA, is directly or indirectly linked to normal telomere function.

### DNA damage and localization of damage- and repair-associated proteins at acrocentric short arms

**Figure 4:**
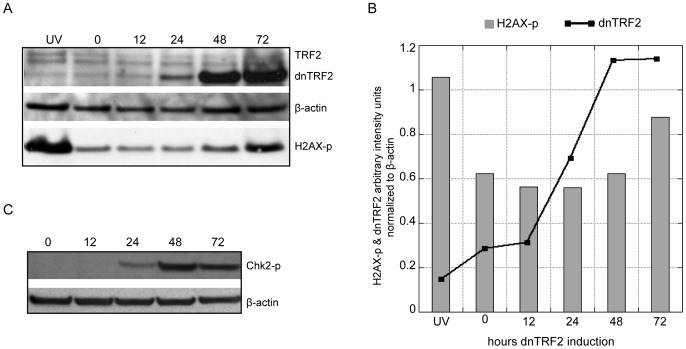
DNA damage markers appear with increased dnTRF2 expression. (A) Immunoblot for TRF2, β-actin (loading control), and H2AX-p on whole cell lysates from UV-treated (∼20 J/m^2^), uninduced/0-hour, 12-hour, 24-hour, 48-hour, and 72-hour dnTRF2-expressing cells. (B) Graphical representation of protein levels measured by arbitrary fluorescence units normalized to β-actin showing increased dnTRF2 protein levels with longer induction periods up to 48 hours. H2AX-p levels increased by 72 hours. (C) Immunoblot for Chk2-p and β-actin showing appearance of phosphorylated Chk2 kinase after 24 hours of dnTRF2 expression.

Unprotected telomeres activate a DNA damage response at chromosome ends. Markers of DNA damage were previously observed both at telomeres and at acrocentric short arms when dnTRF2 was expressed for 48 hours [9]. This timepoint coincided with the appearance of acrocentric fusions, suggesting to us that damage within the short arm was an underlying cause of chromosome fusion in the dnTRF2 assay. We then monitored timing of DNA damage relative to nucleolar disruption and chromosome fusion at distinct intervals after dnTRF2 induction (Figure 4). Western blotting showed a clear increase in dnTRF2 expression between 12 and 48 hours (Figure 4A). Levels of histone H2AX phosphorylated at Serine 139 (H2AX-p), a marker for DNA damage, remained steady until they increased at 72 hours (Figure 4A, 4B). Chk2 phosphorylated Threonine 68 (Chk2-p), a protein kinase that mediates the DNA damage response and protects genome integrity by promoting apoptosis, was detected at 24 hours, and increased as dnTRF2 was expressed for 48–72 hours (Figure 4C). Immunostaining corroborated the increase in H2AX-p and/or Chk2-p associated with isolated nucleoli (Figure S4) or nucleoli in intact nuclei (Figure S5) after 24–48 hours of dnTRF2 expression. These results suggest that in the presence of dnTRF2, nucleolar damage precedes the classical DNA damage response and subsequent fusion at acrocentric short arms.

### dnTRF2 reduces acrocentric and nucleolar enrichment of condensins

**Figure 5:**
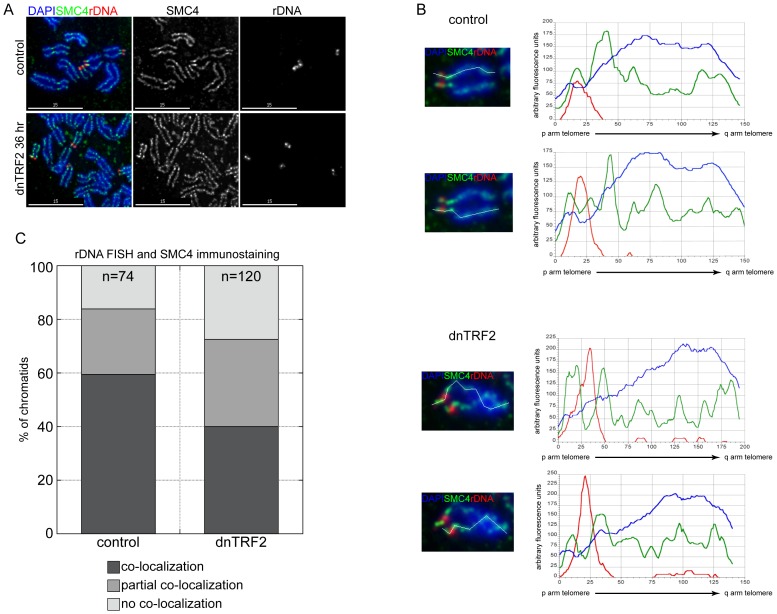
Condensin localization decreases on acrocentric short arms in the presence of dnTRF2. Combined immunostaining-FISH for (A) SMC4 (green) and rDNA (red) on metaphase chromosomes from control and 36 hour dnTRF2-expressing cells. (B) The amount of SMC4 was quantitated by measuring arbitrary fluorescence along the length of the chromosomes and plotting signal intensity as a line plot. A line begins at the p arm (0 on x-axis) and extends to the telomere of the q arm (∼150+ on x-axis). (C) The extent of rDNA and SMC4 co-localization at chromatids of metaphase chromosomes is presented in graphical format. The number of individuals chromatids examined is indicated at the top of each bar. A significant reduction in SMC4 co-localization at rDNA was observed on metaphase chromatids from cells expressing dnTRF2 for 36 hours. Scale bars in (A) are 15 micrometers.

The change in acrocentric short arm morphology suggested alteration of chromosome compaction or condensation. Depletion of condensin has been shown to result in gamma H2AX (H2AX-p) enrichment at centromeres, subtelomeric regions, and rDNA repeats [44]. In dnTRF2 cells, combined immunostaining and FISH on metaphase chromosomes showed that condensin appeared decreased at rDNA or shifted from rDNA onto neighboring repeats (Figure 5). Both SMC2 (not shown) and SMC4 were enriched at nucleoli in control cells, co-localizing with TRF2 (Figure S6A and S6B) and telomeric DNA (not shown). In dnTRF2 expressing cells, less SMC4 and TRF2 were associated with nucleoli (Figure S6C-E). These findings suggest that dnTRF2 results in altered amounts and/or locations of structural proteins on acrocentric short arms.

### Chromatin composition at acrocentric short arms is largely unchanged by dnTRF2

**Figure 6:**
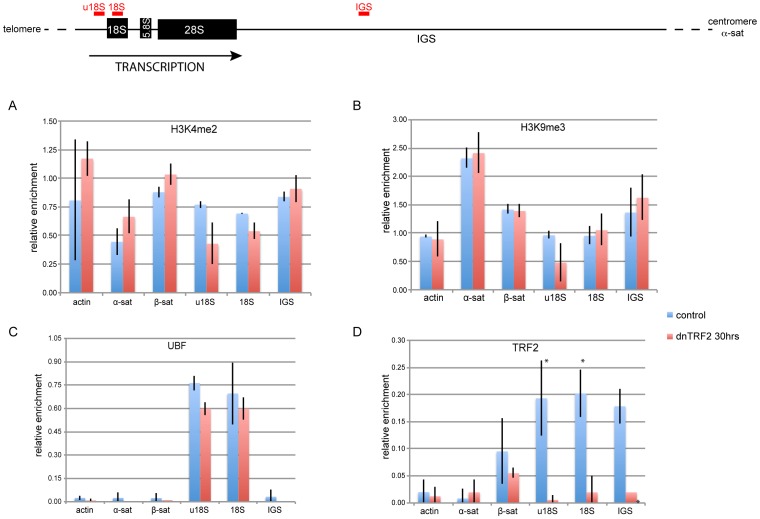
Disruption of TRF2 association, but not open or closed chromatin, at rDNA in the presence of dnTRF2. Chromatin from control/uninduced T19 cells and 30 hour dnTRF2-expressing cells was crosslinked with 1% PFA, sonicated to 100–700 bp, and immunoprecipitated with indicated antibodies: (A) H3K4me2, (B) H3K9me3, (C) UBF, and (D) TRF2. Each bar shows relative enrichment as percentage of input by ChIP-PCR. Actin and alpha satellite are control regions. alpha sat  =  alpha satellite (centromere), β-sat  =  distal beta satellite (located telomeric of rDNA), u18S  =  upstream of 18S rDNA region, 18S = 18S rDNA region, IGS  =  intergenic spacer region in rDNA repeat. Error bars show standard error of the mean. (*) indicates significant difference (*p*<0.001) between control and dnTRF2 ChIP enrichment.

We considered that the abnormal acrocentric short arm morphology that occurred when dnTRF2 and dysfunctional telomeres were present might be linked to chromatin changes. Using chromatin immunoprecipitation (ChIP), we did not detect statistically significant changes in either H3K4me2 (euchromatin) or H3K9me3 (heterochromatin) enrichment at either beta satellite and rDNA regions in control cells and 30-hour dnTRF2 inductions (Figure 6A and 6B). The rDNA transcription factor UBF (upstream binding factor) was also comparably enriched at rDNA (Figure 6C). However, endogenous TRF2 was depleted at three different sites within the rDNA region in dnTRF2 cells - upstream of the 18S region, in the 18S tract, and in the intergenic spacer (IGS) (Figure 6D). These results indicate that in addition to telomeric sequences, TRF2 is also located at rDNA and that dnTRF2 displaces it from acrocentric short arms.

### dnTRF2, NOR morphology, and acrocentric chromosome fusion

**Figure 7:**
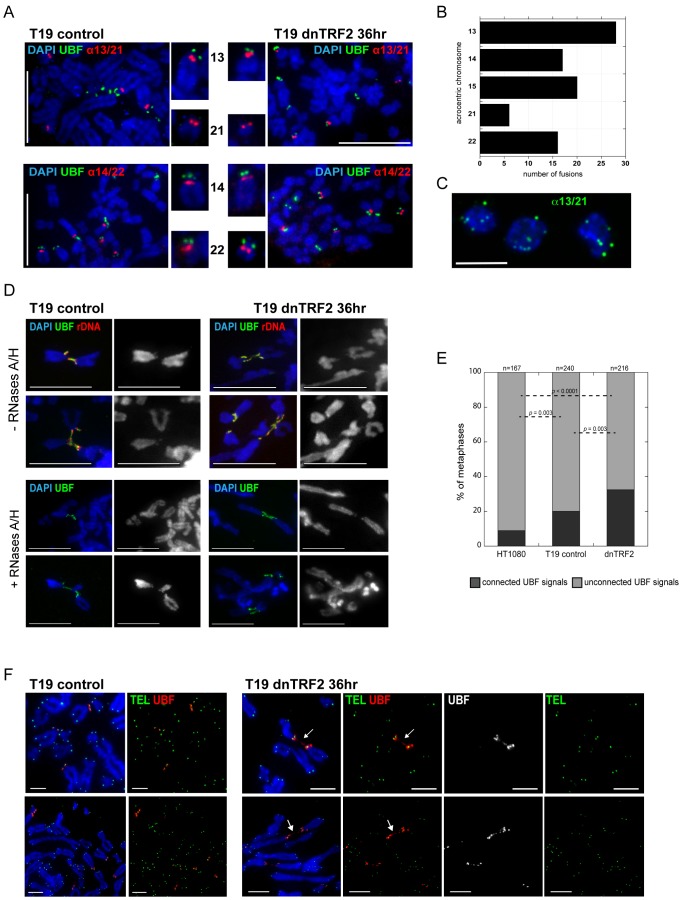
Connections between certain acrocentric short arms during metaphase. (A) Immunostaining-FISH on metaphase chromosomes from T19 control (dnTRF2 uninduced) and T19 dnTRF2 induced (36 hours) cells for UBF (green) and acrocentric alpha satellite DNA (red, alpha satellite 13/21 or alpha satellite 14/22) shows absence of UBF on HSA21. UBF was associated with the short arms of HSA13, HSA14, and HSA22. Scale bars equal 15 micrometers. (B) It was notable that HSA21 did not show UBF immunostaining since this chromosome was involved in the least number of acrocentric fusions when dnTRF2 was transiently expressed for 36 hours. (C) The T19 cells contained an average of 4 copies of HSA13 and 3 copies of HSA21. Isolated nucleoli were hybridized with a FISH probe (green) recognizing the identical alpha satellite sequence that defines the centromeres of HSA13 and HSA21. Nucleoli had an average of 7 FISH signals, suggesting that all copies of HSA13 and HSA21 within the cells were associated with nucleoli. (D) Immunostaining-FISH on metaphase chromosomes revealed the presence and increased persistence of UBF (green) and rDNA (red) within the connections/bridges between acrocentric short arms in dnTRF2-expressing cells. Scale bars equal 15 micrometers. (E) Quantitation of acrocentric bridges/associations. HT1080 control and T19 uninduced control showed fewer UBF mediated acrocentric associations compared to dnTRF2 induced (36 hour) cells. Numbers at the top of each bar represent the total number of metaphases analyzed. Statistical significance (*p* values less than 0.05) was determined by a Chi square test for a 2×2 contingency table. (F) Immunostaining for UBF (red) and FISH with a PNA-TEL probe (Green) demonstrated that connections between acrocentrics in dnTRF-expressing cells did not include telomeric DNA.

Loss of TRF2 at rDNA may not alter chromatin enrichment at rDNA arrays but could affect rDNA transcription. However, using 5FU (5-Fluorouracil) incorporation, quantitative reverse transcriptase PCR (qRT-PCR) or RNA FISH, we did not detect notable differences in overall rDNA transcription between control and dnTRF2-expressing cells (Figure S7). Overall, rDNA transcription did not appear to be decreased or increased by dnTRF2 expression. However, during each cell cycle, only a few of the ten human rDNA arrays are transcriptionally active. These active arrays are associated with UBF and RNA polymerase I transcriptional machinery. UBF sequesters rDNA regions within the nucleus and primes rDNA for binding by the transcriptional machinery [24]. We asked if the presence of UBF at individual rDNA arrays sensitized or protected acrocentric chromosomes from dnTRF2-induced damage and fusion. UBF binding at specific acrocentric chromosomes was visualized using immunofluorescence-FISH (IF-FISH) that, unlike ChIP, allowed each acrocentric to be examined individually. UBF was present at rDNA on HSA13, HSA14 and HSA22 in both control and dnTRF2 cells (Figure 7A), but HSA21 lacked UBF in all cells analyzed. This finding was particularly relevant because we observed that HSA21 was involved in fewer dnTRF2-induced acrocentric fusions [9] (Figure 7B), and suggested the UBF could be a key factor in acrocentric interactions. FISH with a centromere probe recognizing the shared alpha satellite DNA sequence present on HSA13 and HSA21 showed an average of 7 fluorescent signals at nucleoli (Figure 7C). The T19 line has four copies of HSA13 and three copies of HSA21. These results suggest that even though HSA21 lacks UBF, it is associated, at least within the centromere and short arm, with the nucleolus.

Upon closer inspection of individual acrocentrics in dnTRF2 cells, we observed chromosomal connections that stretched between acrocentric chromosomes, such as HSA13, HSA14, and HSA22. They originated at rDNA arrays and were detected with UBF immunostaining and rDNA FISH (Figure 7D and 7E). Control HT1080 cells lacking the dnTRF2 construct, as well as uninduced T19 lines, exhibited very few UBF/rDNA inter-acrocentric connections (Figure 7D). The composition of these chromosomal bridges appeared to be both DNA and protein. They did not appear to contain RNA, since they remained between acrocentrics, even after treatment of cells with RNase A and RNase H (Figure 7C). Since chromosome ends are destabilized in the dnTRF2 assay and eventually lead to end-fusions [10], it was possible that the bridges contained DNA originating from the telomere through the rDNA. However, FISH with a telomere probe showed that telomeric DNA appeared as fluorescent dots at chromosome ends and was not present in the bridges between the acrocentrics (Figure 7F). These results indicate that acrocentric short arms are physically linked to one another via UBF-coated rDNA bridges that persist and are more numerous in the presence of dnTRF2.

## Discussion

The nucleolus is crucial to cell proliferation, with a primary role in processing and assembling ribosomes. Beyond this fundamental role, further investigations have revealed a more dynamic character – with a diversity of proteins passing in and out of the nucleolus [27], [45]. It is currently unclear what controls or drives chromosomal and protein movement in and out of the nucleolus during the cell cycle and/or in response to stress. Here we have shown that TRF2, a central component of the proteinaceous structure at the telomere, plays an extra-telomeric role in nucleolar and acrocentric NOR stability.

TRF2 was previously shown to localize with nucleoli and UBF in a cell cycle dependent manner [46]. Other telomere-associated proteins have been observed in the nucleolus, including TRF1, WRN helicase, Dyskerin, and BLM helicase [47]–[50]. Our ChIP experiments have supported an association between TRF2 and rDNA that is lost upon dnTRF2 expression. The presence of endogenous TRF2 at rDNA is likely due to protein-protein interactions with rDNA-associated proteins, since TRF2 has a specific affinity for double stranded TTAGGG repeats [3]. Nevertheless, TRF2 can bind to DNA junctions, like replication forks or Holliday junctions that lack telomeric sequence, and at non-telomeric double-stranded breaks [51], [52]. Our results support a novel association of TRF2 at the rDNA array, but it remains unclear if TRF2 interacts directly with the nucleolar DNA or through associations with other nucleolar or chromosomal proteins. ChIP-sequencing data has identified TRF2 at non-telomeric sites and in proximity to genes, as well as at interstitial telomere repeats, strengthening the argument for extra-telomeric roles for TRF2 [53], [54]. Our data suggest that there are interactions between TRF2 and UBF and/or condensin components that regulate NOR morphology and acrocentric short arm stability. Future biochemical studies with robust antibodies will be important for establishing protein-protein interactions that are disrupted by mutant TRF2.

Loss of TRF2 function through either dnTRF2 expression or RNAi knockdown, or disruption of telomere function by zeocin-induced DNA damage, resulted in drastic changes to nucleolar structure. There is wide variation in nucleolar response to stress and DNA damage, although it is generally considered the hub for directing the stress response [27]. For example, in response to cellular UV exposure, the nucleolus segments into nucleolar caps [55]. In our studies, the nucleolus was sensitive to even low levels of dnTRF2 expression as early as 12 hours after induction, as exhibited by nucleolar fragmentation and necklace formation. Such fragmentation mimicked the nucleolar morphology that has been described when RNA polymerase II is inhibited [56]. In our studies of dnTRF2 expression, nucleolar dysmorphology preceded the appearance of acrocentric and telomeric DNA damage denoted by H2AX-p. It also occurred prior to chromosome fusion. Whether nucleolar reorganization in this context is an early stress indicator that reflects NOR sensitivity or is simply a response to limited damage detection at some telomeres remains to be determined. TRF2 at telomeres represses the activation of the ATM kinase pathway, thereby preventing the cell from recognizing telomeres as broken DNA strands [7]. Thus, TRF2 might initiate or influence the DNA damage response pathway via the nucleolus.

The NOR is key to nucleoli formation that is induced by the binding of UBF to rDNA arrays. This is followed by sequestration of NORs, and consequently NOR-bearing chromosomes, to subdomains of the nucleus. The overall morphology of the NOR is distinctive during mitosis as the region is ten-fold less compact, giving the acrocentric short arm what only appears to be a constricted appearance [57]. UBF drives this process, as well as recruiting transcriptional machinery to transcribe the ribosomal genes [24]. Human cells contain an overabundance of rDNA repeats, and only some NORs are transcriptionally active. Changes in nucleolar architecture detected by FISH may be attributed to alterations in the organization, chromatin compaction, and DNA-DNA interactions within the rDNA. In support of a chromatin-remodeling model, we observed nucleolar co-localization of TRF2 and SMC4, a subunit of the condensin complex. In yeast, condensin is required for rDNA segregation and stability following starvation or rapamycin treatment [58], [59]. A recent investigation in human cells showed H2AX-p localization to rDNA repeats following SMC2 knockdown [44]. Without adequate condensin recruitment or maintainence in times of cellular stress, this repetitive and highly transcribed region becomes unstable.

It is particularly notable that UBF did not disappear from rDNA arrays in the presence of dnTRF2. UBF is an important mediator of rDNA transcription dynamics as well as NOR morphology. UBF recruits RNA polymerase I transcriptional machinery to ribosomal genes through protein-protein interactions, but these factors dwell for only seconds on the rDNA. UBF binding to NORs, even those that are transcriptionally inert, induces distinctive undercondensed, open chromatin morphology [60]. Considering that UBF creates and maintains the under-condensed state of NORs, the change in acrocentric short arm compaction during dnTRF2 expression may be due relocation of condensin from specific regions of the short arm and/or the nucleolus. The non-random acrocentric fusions that occur when dnTRF2 is expressed most likely represent repair of DNA damage that arises as the short arms that are physically linked by UBF become more stretched. About half of induced acrocentric fusions are not telomere-telomere fusions and in fact lack one or more additional repetitive sequences immediately adjacent to telomeres [9]. These previous findings, combined with our observations here that acrocentric short arms are tethered to each other, suggest that repair leading to acrocentric fusions occurs by NHEJ between closely located chromosomes. As telomere damage persists in the presence of dnTRF2, other chromosome fusions eventually occur. These subsequent fusions probably involve chromosomes that are closely located in the nucleus but are not physically connected like the acrocentrics. This model is strengthened by our observation that HSA21 lacked UBF binding and eventually became involved in fusions that occurred days after acrocentric short arms that were tethered by rDNA and UBF connections had already fused [9].

Here we report that several approaches used to disrupt telomere function all result in increased fusions between human acrocentric chromosome and indicating that acrocentric short arms, in particular the rDNA, are especially prone to instability following telomere disruption. Our findings reveal a novel and unexpected relationship between TRF2 protein function at the nucleolus and NOR stability, potentially mediated through UBF and other chromosomal structural proteins. These studies also suggest that acrocentric chromosomes reside in defined nuclear domains that prime them for their non-random interactions. This has significant implications for understanding formation of recurrent chromosome rearrangements, like Robertsonian translocations, that are associated with birth defects and cancer. These results provide a framework for future studies exploring in more detail the protein-protein and protein-DNA connections that link acrocentric short arms to one another and to the nucleolus. Such findings could lead to more sensitive methods to capture, or prevent, formation of specific acrocentric chromosome fusions in humans.

## Supporting Information

Figure S1 **Nucleolar changes occur upon TRF2 RNAi knockdown.** (A) Immunostaining for TRF2 (green) and fibrillarin (red) of HT1080 nuclei from cells transfected with empty vector (control) and shRNA directed against TRF2. Cells were selected for short hairpin constructs with puromycin for ∼1 week before immunostaining. TRF2 and fibrillarin immunostaining in knockdown cells showed that much of TRF2 had been depleted, although some residual remained. Fibrillarin staining showed the disruption (unraveled appearance) of the nucleolus that mirrored the change in morphology caused by dnTRF2. (B) Quantitation of TRF2 immunostaining in control and TRF2 knockdown cells showed that the intensity of detectable TRF2 decreased significantly when TRF2 was depleted. (C) Quantitation of the percentage of nucleoli that showed altered morphology, specifically loss of punctate staining and unraveled, necklace appearance. This staining was comparable to that seen with dnTRF2 expression (see Figure 2).
(TIF)

Figure S2 **Nucleolar changes associated with prolonged dnTRF2 expression.** Immunostaining of the nucleolus and dnTRF2 (FLAG) after dnTRF2 expression for 36–45 hours. (A) Fibrillarin (green) staining in control cells was localized into more compact structures but appeared more scattered within the nucleus after prolonged dnTRF2 expression. (B) Combined fibrillarin (green), B23 (red), and dnTRF2 (FLAG - blue) immunostaining revealed notable morphological changes in the nucleoli in the presence of dnTRF2. Single channel gray-scale images are shown to emphasize immunostaining for specific proteins. Scale bars are 5 microns.
(TIF)

Figure S3 **Acrocentric short arm repeat morphology after dnTRF2 expression.** FISH on whole nuclei from control (T19 uninduced) and T19 dnTRF2-expressing cells induced for 24 hours and 3 days. Single channel images for each FISH probe are shown under the merged DAPI-FISH probe image. (A) FISH for β-satellite DNA (red) showed that FISH signals were more scattered throughout the nucleus but stayed relatively punctate after dnTRF2 expression for up to 3 days. (B) FISH for sat III acrocentric short arm repeat (green) showed a slight, though not significant, increase in dispersion with dnTRF2 expression. (C) FISH for centromeric alpha satellite using a CENP-B box PNA probe (green). FISH signals did not show any detectable changes between control and dnTRF2 cells. (D) Quantitation of short arm DNA morphology in multiple time increments (12-hour, 24-hour, 36-hour dnTRF2 inductions) illustrates that with persistent dnTRF2 expression, normally punctate FISH signals (grey bars) decreased while the percentage of nuclei exhibiting abnormal FISH signals for 18S rDNA (red bars), complete rDNA array (orange bars), and satellite III (green bars) increase. The number of nuclei evaluated at each time point is indicated at the bottom of each bar. Significance is illustrated by *p*-values between control and dnTRF2-expression time points.
(TIF)

Figure S4 **Markers of DNA damage are associated with isolated nucleoli in dnTRF2-expressing cells.** Immunostaining of isolated nucleoli, identified with fibrillarin antibodies (red), from control and dnTRF2-induced cells showed increased association of DNA damage markers (A) H2AX-p (green), (C) Chk2-p (green), and (E) ATM-p (green). (B, D, F) The total fluorescence intensity/integrated density of antibody signals for the DNA damage markers against mean fibrillarin signal intensity was measured on individual nucleoli and plotted in box plots. Statistically significant differences (*p*<0.05) were calculated using a Student *t*-test.
(TIF)

Figure S5 **DNA damage in whole nuclei accumulates at nucleoli in the presence of dnTRF2.** Immunostaining and FISH on three-dimensionally preserved nuclei from control and dnTRF2-expressing cells showed an increase in the association of gamma H2AX (red) at nucleoli after 48 hour dnTRF expression. Fibrillarin (green) was used to identify nucleoli. Pearson Coefficient of Correlation was used to quantitate the amount of co-localization between fibrillarin and gamma H2AX. The average coefficient was higher (0.38) for dnTRF2-expressing cells compared to controls (0.23), suggesting more association of DNA damage at nucleoli.
(TIF)

Figure S6 **Condensin localization at isolated nucleoli decreases with dnTRF2 expression.** (A) The condensin subunit SMC4 (green) localizes in a punctate pattern at isolated nucleoli marked by immunostaining with fibrillarin (FIB). (B) SMC4 (green) shows some co-localization with TRF2 (red). Scale bars in all panels throughout the figure are 5 micrometers. (C) Extent of SMC4 (green) and TRF2 (red) co-localization in control cells indicated with a plot showing the Pearson coefficient of correlation. Higher coefficients (approaching 1) denote a greater degree of overlap with SMC4 (AFU =  arbitrary fluorescence unit). (D) Decreased co-localization of TRF2 with SMC4 after 30 hours of dnTRF2 expression is indicated by a lower coefficient. (E) Pearson coefficient of correlation (SMC4 x TRF2) was measured for multiple control nucleoli (grey) and dnTRF2 30-hour nucleoli (black). The scatterplot shows a significant decrease in overlap with dnTRF2 expression.
(TIF)

Figure S7 **Global rDNA transcription is unchanged during nucleolar disruption.** (A) Ongoing transcription in nuclei was visualized by detecting 5FU (green) incorporation into nascent RNA after 30 minutes of 5FU incubation. 5FU signal mainly localized to nucleoli and tracked with the unraveling fibrillarin (red). Uninduced control cells are in the top row, followed by 24-hour, 48-hour, and 72-hour dnTRF2-expressing cells. The bottom two rows show cells treated with Actinomycin D (ActD), an inhibitor of RNA transcription. Scale bars equal 5 micrometers. (B) qRT-PCR analysis was carried out for 45S pre-ribosomal RNA (blue) and 28S ribosomal RNA (green) transcript levels in control, 12-hour, 24-hour, 48-hour, and 72-hour dnTRF2-expressing cells, as well as serum starved (ss), 30 minute ActD, and 3 hour ActD-treated cells. Results are presented as fold change in transcription (based on Ct values) from T19 (uninduced) control cells. (C) 18S RNA FISH signals (green) co-localize with fibrillarin (red) in nuclei from control and T19 cells expressing dnTRF2 for 3 days. Scale bars equal 30 micrometers.
(TIF)
