# Supplementary figures and images for: Nucleolar Organization, Ribosomal DNA Array Stability, and Acrocentric Chromosome Integrity Are Linked to Telomere Function
Source: PLoS One. 2014 Mar 24;9(3):e92432. doi: 10.1371/journal.pone.0092432 (PMC3963894; doi:10.1371/journal.pone.0092432)

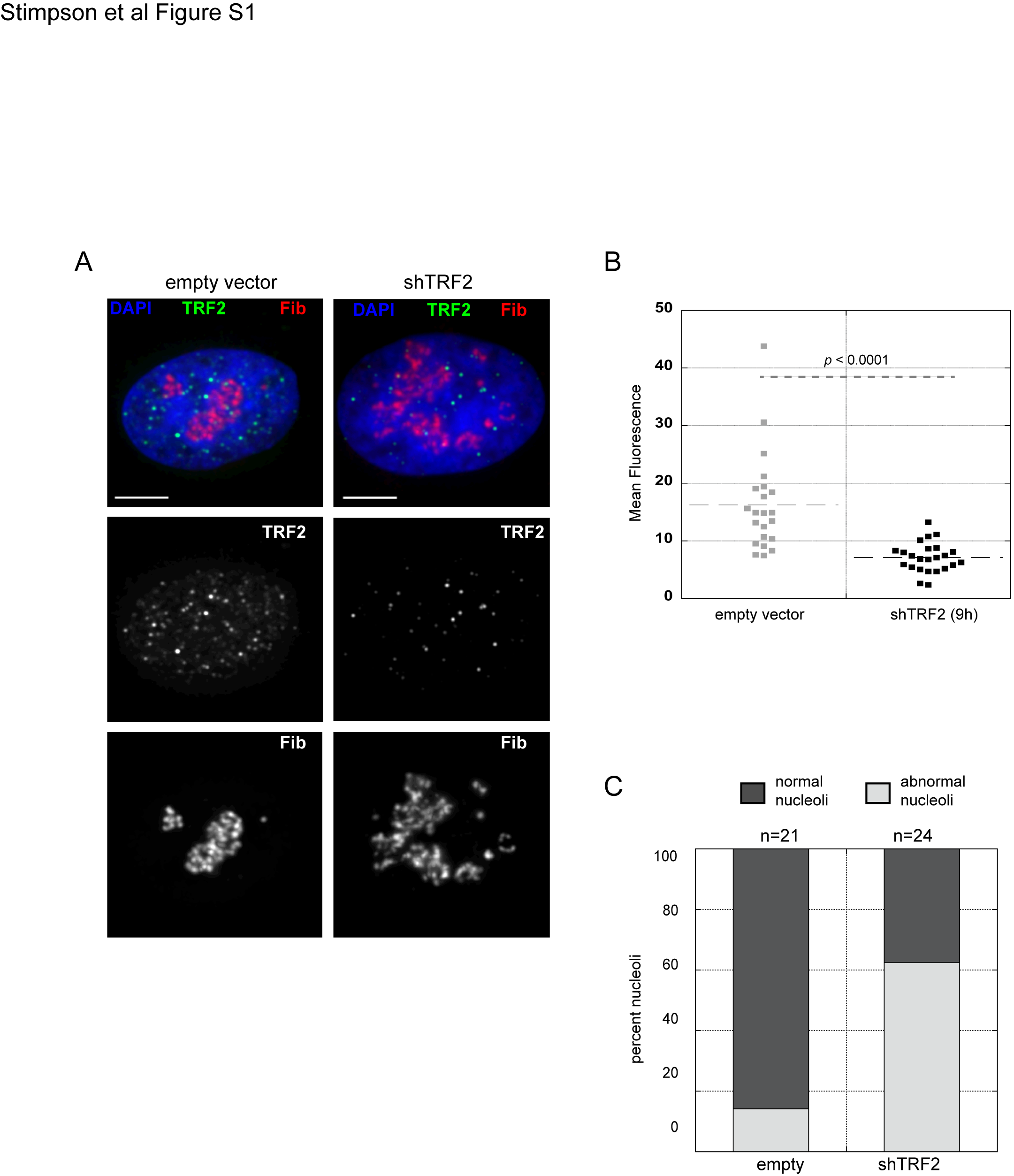

Supplement: Figure S1 — Nucleolar changes occur upon TRF2 RNAi knockdown. (A) Immunostaining for TRF2 (green) and fibrillarin (red) of HT1080 nuclei from cells transfected with empty vector (control) and shRNA directed against TRF2. Cells were selected for short hairpin constructs with puromycin for ∼1 week before immunostaining. TRF2 and fibrillarin immunostaining in knockdown cells showed that much of TRF2 had been depleted, although some residual remained. Fibrillarin staining showed the disruption (unraveled appearance) of the nucleolus that mirrored the change in morphology caused by dnTRF2. (B) Quantitation of TRF2 immunostaining in control and TRF2 knockdown cells showed that the intensity of detectable TRF2 decreased significantly when TRF2 was depleted. (C) Quantitation of the percentage of nucleoli that showed altered morphology, specifically loss of punctate staining and unraveled, necklace appearance. This staining was comparable to that seen with dnTRF2 expression (see Figure 2). (TIF) [file pone.0092432.s001.tif]

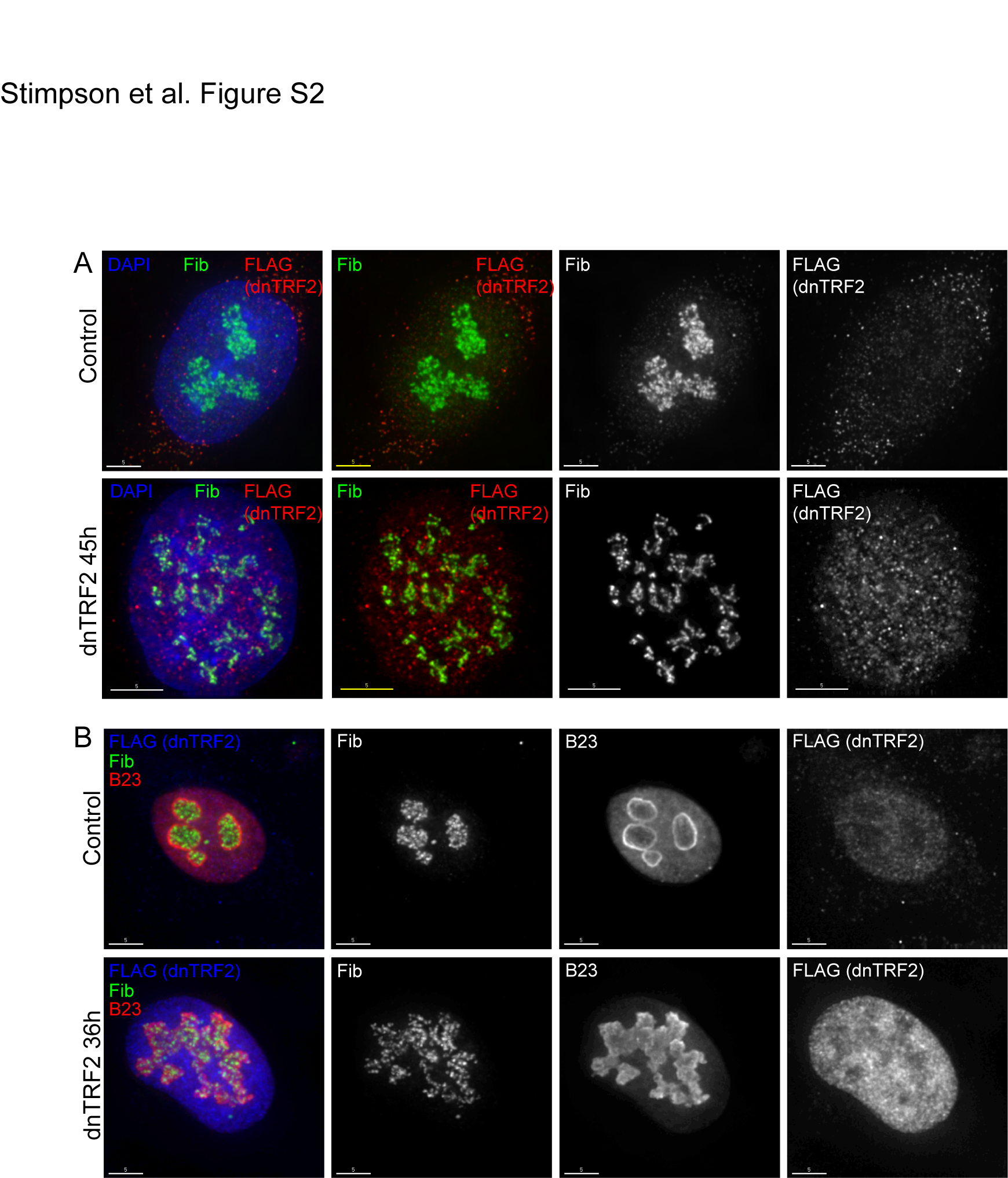

Supplement: Figure S2 — Nucleolar changes associated with prolonged dnTRF2 expression. Immunostaining of the nucleolus and dnTRF2 (FLAG) after dnTRF2 expression for 36–45 hours. (A) Fibrillarin (green) staining in control cells was localized into more compact structures but appeared more scattered within the nucleus after prolonged dnTRF2 expression. (B) Combined fibrillarin (green), B23 (red), and dnTRF2 (FLAG - blue) immunostaining revealed notable morphological changes in the nucleoli in the presence of dnTRF2. Single channel gray-scale images are shown to emphasize immunostaining for specific proteins. Scale bars are 5 microns. (TIF) [file pone.0092432.s002.tif]

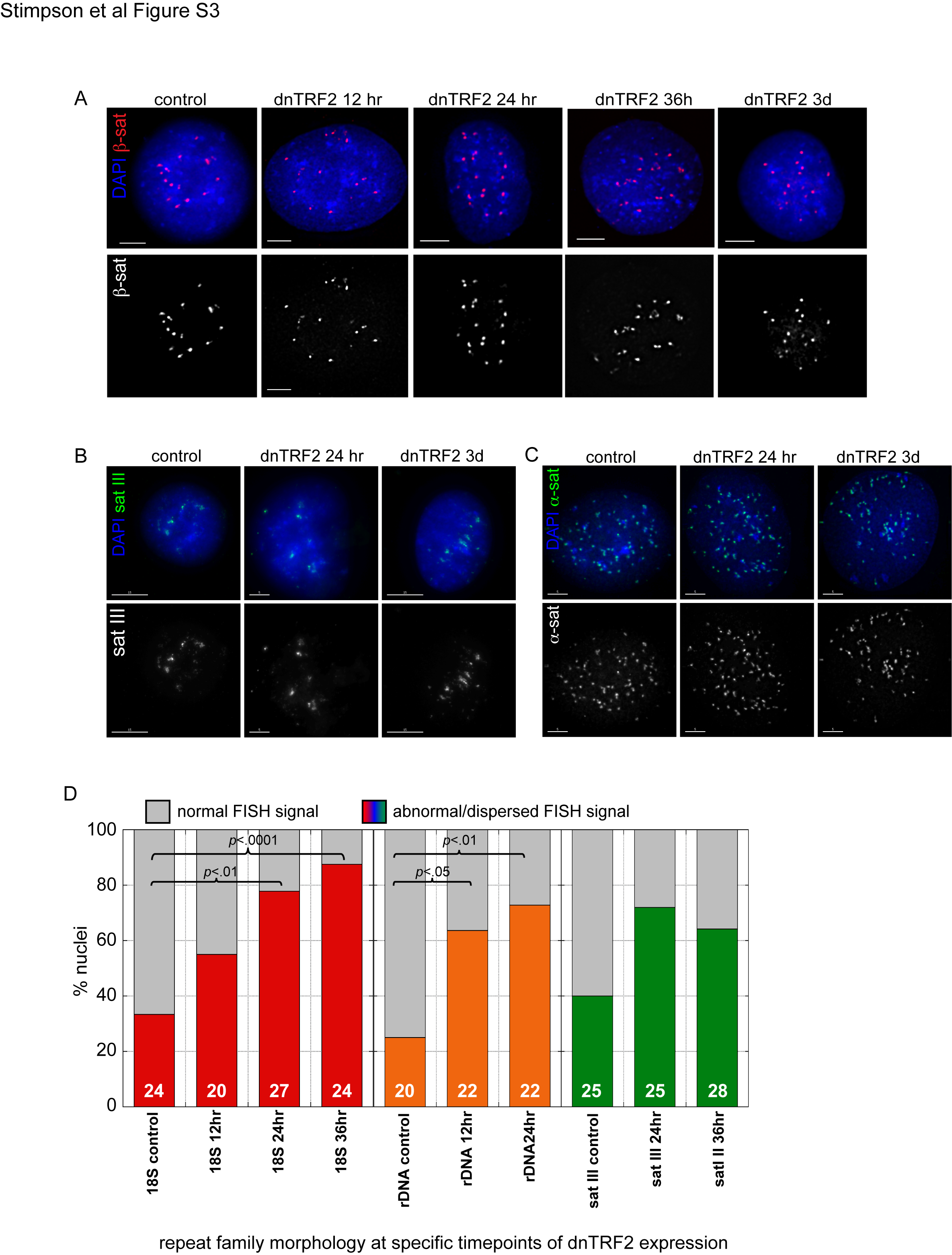

Supplement: Figure S3 — Acrocentric short arm repeat morphology after dnTRF2 expression. FISH on whole nuclei from control (T19 uninduced) and T19 dnTRF2-expressing cells induced for 24 hours and 3 days. Single channel images for each FISH probe are shown under the merged DAPI-FISH probe image. (A) FISH for β-satellite DNA (red) showed that FISH signals were more scattered throughout the nucleus but stayed relatively punctate after dnTRF2 expression for up to 3 days. (B) FISH for sat III acrocentric short arm repeat (green) showed a slight, though not significant, increase in dispersion with dnTRF2 expression. (C) FISH for centromeric alpha satellite using a CENP-B box PNA probe (green). FISH signals did not show any detectable changes between control and dnTRF2 cells. (D) Quantitation of short arm DNA morphology in multiple time increments (12-hour, 24-hour, 36-hour dnTRF2 inductions) illustrates that with persistent dnTRF2 expression, normally punctate FISH signals (grey bars) decreased while the percentage of nuclei exhibiting abnormal FISH signals for 18S rDNA (red bars), complete rDNA array (orange bars), and satellite III (green bars) increase. The number of nuclei evaluated at each time point is indicated at the bottom of each bar. Significance is illustrated by p-values between control and dnTRF2-expression time points. (TIF) [file pone.0092432.s003.tif]

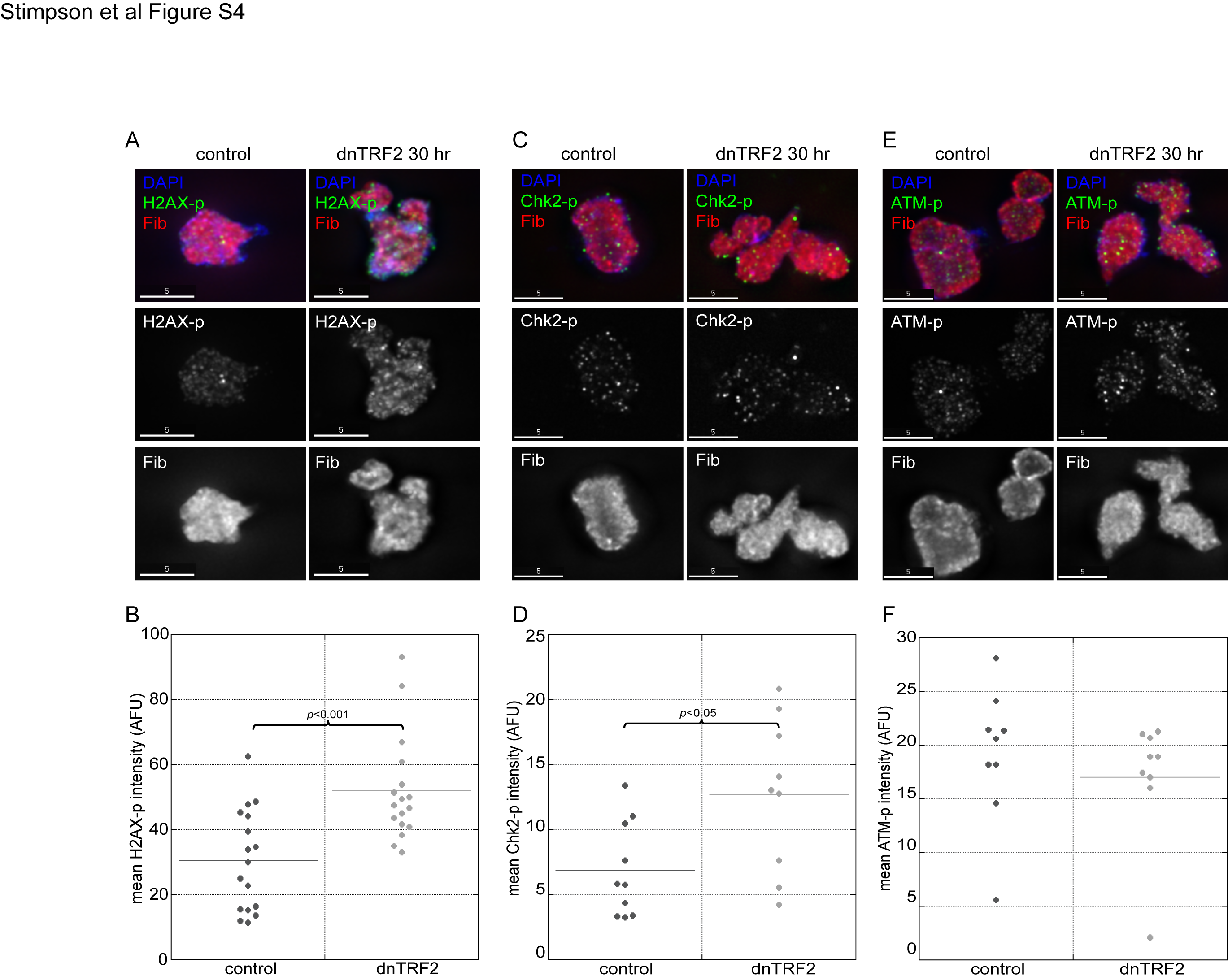

Supplement: Figure S4 — Markers of DNA damage are associated with isolated nucleoli in dnTRF2-expressing cells. Immunostaining of isolated nucleoli, identified with fibrillarin antibodies (red), from control and dnTRF2-induced cells showed increased association of DNA damage markers (A) H2AX-p (green), (C) Chk2-p (green), and (E) ATM-p (green). (B, D, F) The total fluorescence intensity/integrated density of antibody signals for the DNA damage markers against mean fibrillarin signal intensity was measured on individual nucleoli and plotted in box plots. Statistically significant differences (p<0.05) were calculated using a Student t-test. (TIF) [file pone.0092432.s004.tif]

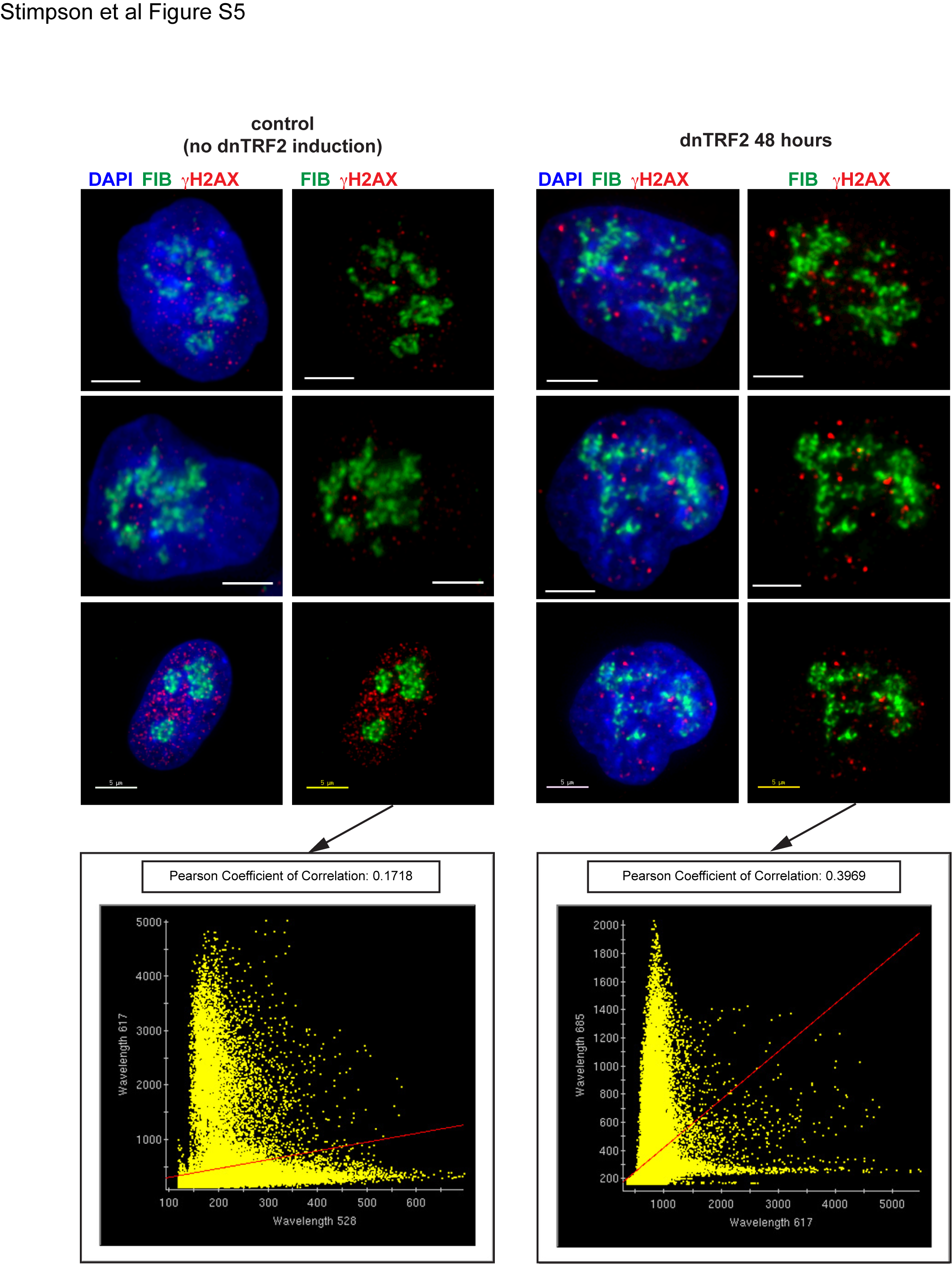

Supplement: Figure S5 — DNA damage in whole nuclei accumulates at nucleoli in the presence of dnTRF2. Immunostaining and FISH on three-dimensionally preserved nuclei from control and dnTRF2-expressing cells showed an increase in the association of gamma H2AX (red) at nucleoli after 48 hour dnTRF expression. Fibrillarin (green) was used to identify nucleoli. Pearson Coefficient of Correlation was used to quantitate the amount of co-localization between fibrillarin and gamma H2AX. The average coefficient was higher (0.38) for dnTRF2-expressing cells compared to controls (0.23), suggesting more association of DNA damage at nucleoli. (TIF) [file pone.0092432.s005.tif]

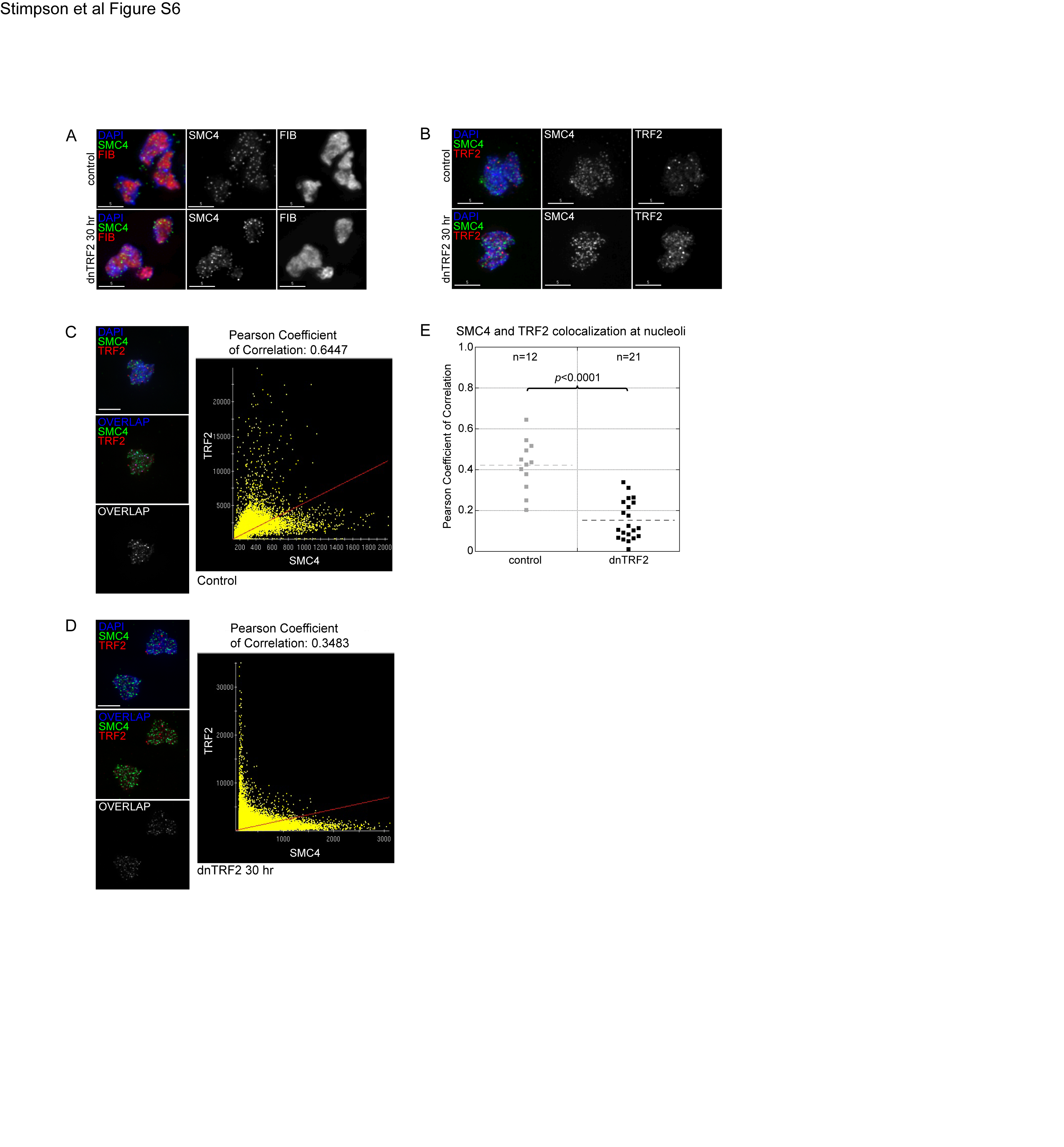

Supplement: Figure S6 — Condensin localization at isolated nucleoli decreases with dnTRF2 expression. (A) The condensin subunit SMC4 (green) localizes in a punctate pattern at isolated nucleoli marked by immunostaining with fibrillarin (FIB). (B) SMC4 (green) shows some co-localization with TRF2 (red). Scale bars in all panels throughout the figure are 5 micrometers. (C) Extent of SMC4 (green) and TRF2 (red) co-localization in control cells indicated with a plot showing the Pearson coefficient of correlation. Higher coefficients (approaching 1) denote a greater degree of overlap with SMC4 (AFU = arbitrary fluorescence unit). (D) Decreased co-localization of TRF2 with SMC4 after 30 hours of dnTRF2 expression is indicated by a lower coefficient. (E) Pearson coefficient of correlation (SMC4 x TRF2) was measured for multiple control nucleoli (grey) and dnTRF2 30-hour nucleoli (black). The scatterplot shows a significant decrease in overlap with dnTRF2 expression. (TIF) [file pone.0092432.s006.tif]

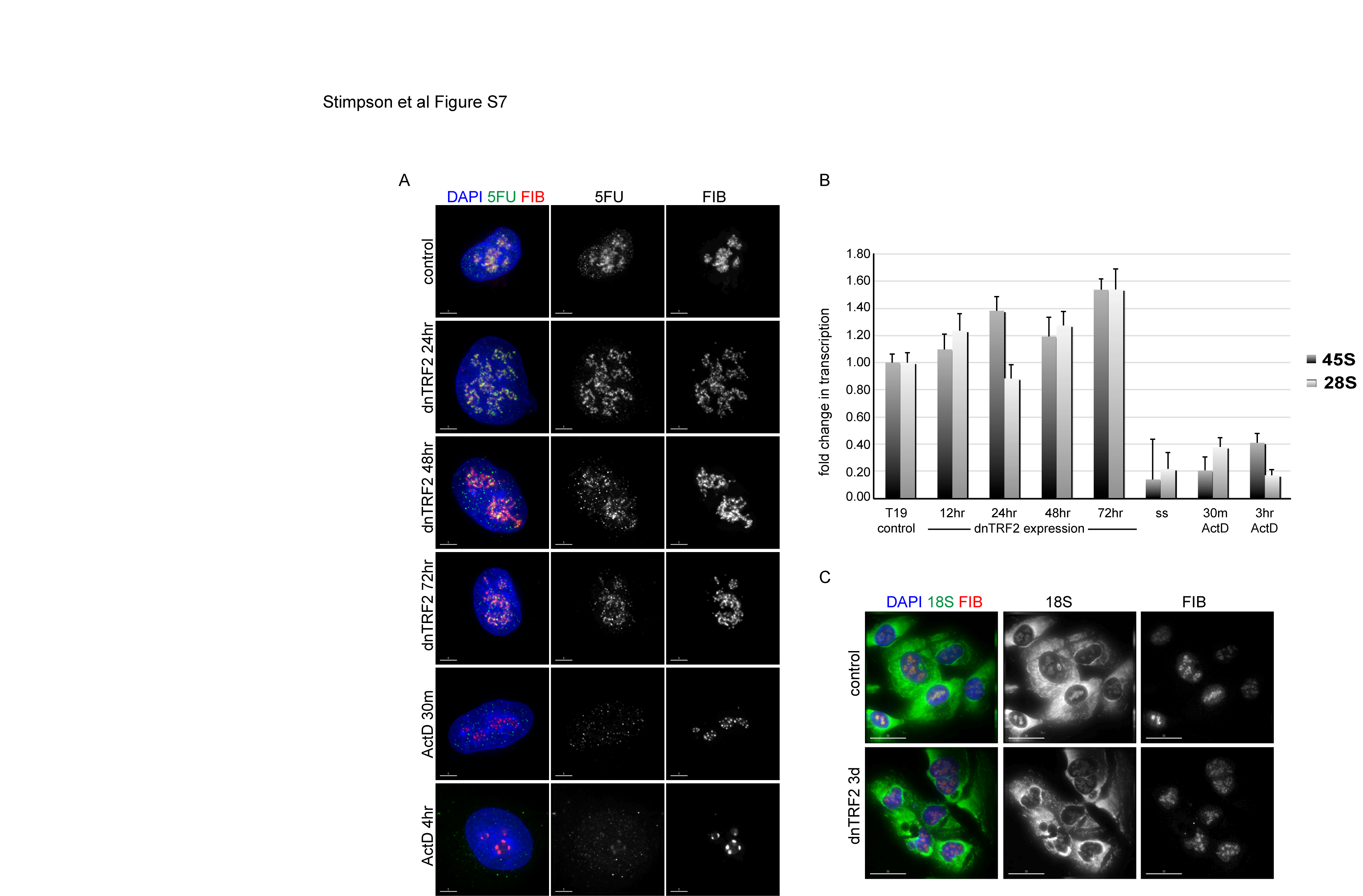

Supplement: Figure S7 — Global rDNA transcription is unchanged during nucleolar disruption. (A) Ongoing transcription in nuclei was visualized by detecting 5FU (green) incorporation into nascent RNA after 30 minutes of 5FU incubation. 5FU signal mainly localized to nucleoli and tracked with the unraveling fibrillarin (red). Uninduced control cells are in the top row, followed by 24-hour, 48-hour, and 72-hour dnTRF2-expressing cells. The bottom two rows show cells treated with Actinomycin D (ActD), an inhibitor of RNA transcription. Scale bars equal 5 micrometers. (B) qRT-PCR analysis was carried out for 45S pre-ribosomal RNA (blue) and 28S ribosomal RNA (green) transcript levels in control, 12-hour, 24-hour, 48-hour, and 72-hour dnTRF2-expressing cells, as well as serum starved (ss), 30 minute ActD, and 3 hour ActD-treated cells. Results are presented as fold change in transcription (based on Ct values) from T19 (uninduced) control cells. (C) 18S RNA FISH signals (green) co-localize with fibrillarin (red) in nuclei from control and T19 cells expressing dnTRF2 for 3 days. Scale bars equal 30 micrometers. (TIF) [file pone.0092432.s007.tif]
